# Appropriateness of Imaging for Low-Risk Prostate Cancer—Real World Data from the Pennsylvania Urologic Regional Collaboration (PURC)

**DOI:** 10.3390/curroncol31080354

**Published:** 2024-08-20

**Authors:** Raidizon Mercedes, Dennis Head, Elizabeth Zook, Eric Eidelman, Jeffrey Tomaszewski, Serge Ginzburg, Robert Uzzo, Marc Smaldone, John Danella, Thomas J. Guzzo, Daniel Lee, Laurence Belkoff, Jeffrey Walker, Adam Reese, Mihir S. Shah, Bruce Jacobs, Jay D. Raman

**Affiliations:** 1 Department of Urology, Penn State College of Medicine, Hershey, PA 17033, USA; 2 Department of Urology, Cooper University Health Care, Camden, NJ 08103, USA; 3 Department of Urology, Einstein Healthcare Network, Philadelphia, PA 19141, USA; 4 Department of Urology, Fox Chase Cancer Center, Philadelphia, PA 19111, USA; 5 Department of Urology, Geisinger Health, Danville, PA 17822, USA; 6 Department of Urology, University of Pennsylvania Health System, Philadelphia, PA 19104, USA; 7 MidLantic Urology, Bala Cynwyd, PA 19008, USA; 8 Department of Urology, Temple University Hospital, Philadelphia, PA 19140, USA; 9 Department of Urology, Thomas Jefferson University Hospital, Philadelphia, PA 19107, USA; mihir.shah@jefferson.edu; 10 Department of Urology, University of Pittsburgh Medical Center, Pittsburgh, PA 15219, USA

**Keywords:** over-imaging, redundant, risk stratification, CT, MRI, PET

## Abstract

Imaging for prostate cancer defines the extent of disease. Guidelines recommend against imaging low-risk prostate cancer patients with a computed tomography (CT) scan or bone scan due to the low probability of metastasis. We reviewed imaging performed for men diagnosed with low-risk prostate cancer across the Pennsylvania Urologic Regional Collaborative (PURC), a physician-led data sharing and quality improvement collaborative. The data of 10 practices were queried regarding the imaging performed in men diagnosed with prostate cancer from 2015 to 2022. The cohort included 13,122 patients with 3502 (27%) low-risk, 2364 (18%) favorable intermediate-risk, 3585 (27%) unfavorable intermediate-risk, and 3671 (28%) high-risk prostate cancer, based on the AUA guidelines. Amongst the low-risk patients, imaging utilization included pelvic MRI (59.7%), bone scan (17.8%), CT (16.0%), and PET-based imaging (0.5%). Redundant imaging occurred in 1022 patients (29.2%). There was variability among the PURC sites for imaging used in the low-risk patients, and iterative education reduced the need for CT and bone scans. Approximately 15% of low-risk patients had staging imaging performed using either a CT or bone scan, and redundant imaging occurred in almost one-third of men. Such data underscore the need for continued guideline-based education to optimize the stewardship of resources and reduce unnecessary costs to the healthcare system.

## 1. Introduction

Imaging studies aim to assess the extent of disease locally and identify any nodal or distant metastases, which helps guide treatment decisions. Newly diagnosed prostate cancer patients are stratified into risk groups that consider the likelihood of metastatic disease to help dictate imaging and patient management. For asymptomatic patients with low-risk prostate cancer, defined by the American Urological Association (AUA) guidelines, the probability of distant metastasis is low (<1.5%) [1,2,3,4]. The current AUA guidelines recommend that clinicians should not routinely perform abdomino-pelvic computed tomography (CT) or bone scans in asymptomatic patients with low- or intermediate-risk prostate cancer, and those imaging types should be reserved for patients with high-risk disease [4]. Additionally, the current AUA guidelines recommend that patients with prostate cancer who have a high risk of metastatic disease and negative conventional imaging may obtain prostate-specific membrane antigen positron emission tomography (PSMA PET) to evaluate for metastatic disease [5]. This guideline statement is based on expert opinion due to the lack of prospective evidence, so molecular imaging may also be obtained at the discretion of the treating physician without obtaining a negative conventional imaging first [5]. In the context of low-risk prostate cancer, magnetic resonance imaging (MRI) has been recognized as a valuable tool as it can help in determining the appropriate treatment approach for low-risk prostate cancer patients and aid in both radiotherapy and surgical planning [6].

The AUA guidelines align with the Choosing Wisely Campaign, launched in 2012, that aimed to facilitate the decision between healthcare providers and patients on unnecessary medical tests, treatments, and procedures. This campaign sought to decrease inappropriate staging imaging for men with low-risk prostate cancer and encourage the stewardship of resources [7]. Routine imaging tests like CTs, MRIs, or bone scans for early-stage low-risk prostate cancer do not offer clinical benefits but come with significant costs [8]. Both the American Society of Clinical Oncology and the AUA have stressed the importance of reducing inappropriate imaging for low-risk prostate cancer within the Choosing Wisely Campaign in order to cut down on unnecessary imaging, decrease healthcare resource overuse, and enhance quality of care [9]. 

In this study, we reviewed imaging performed for men diagnosed with low-risk prostate cancer across a large regional quality collaborative. Our primary objective was to evaluate real-world data concerning the use of imaging modalities, specifically MRIs, CTs, bone scans, and PSMA PET scans, in this patient population. By examining trends over time, we aimed to understand how imaging practices have evolved and whether education can aid in compliance with current guidelines. 

## 2. Materials and Methods

The Pennsylvania Urologic Regional Collaborative (PURC) is a prospective quality improvement collaborative of diverse urology practices across Pennsylvania and New Jersey, with the goal of improving the quality of care provided during the diagnosis, management, and treatment of patient with prostate cancer or undergoing prostate biopsy. This study was performed with a dataset that was obtained through a shared data use agreement with PURC. At the time of writing this manuscript, PURC consisted of 13 practices and 170 physicians with data on over 22,000 men with prostate cancer. 

The PURC data registry was queried for patients over the age of 18 who were diagnosed with low-risk prostate cancer, according to the AUA guidelines, between the years 2015 and 2022. For this study, ten practices had data available for query regarding the imaging performed. We excluded men who were diagnosed with prostate cancer but had no imaging data. 

The dataset obtained from the PURC registry contained detailed information for each patient entered into the system by data abstractors. AUA risk stratification for each patient was calculated in PURC. The exported data were cleaned in Stata 18 statistical software to optimize fidelity and accuracy. Summary statistics and summary tables for low-risk prostate cancer patients were analyzed in Stata, then exported into Microsoft Excel for graphics generation. 

Our primary outcome measure was the type of imaging modality (MRI, CT scan, bone scan, PSMA PET scan) obtained by the patient with low-risk prostate cancer. Additionally, we assessed the occurrence of redundant imaging, defined as patients receiving multiple imaging studies. We analyzed the distribution of imaging modalities within our cohort of interest. Secondary analyses investigated the variability of imaging practices across the 10 participating sites. Furthermore, we examined temporal trends in the utilization of CT and bone scans to assess changes in imaging practices over time. 

## 3. Results

**Figure 1:**
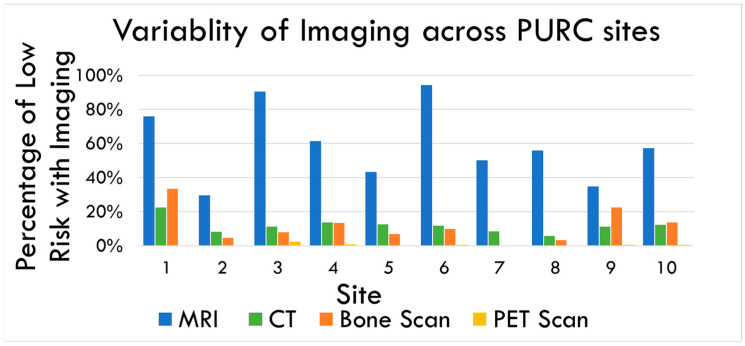
Distribution of imaging modalities among low-risk prostate cancer patients across the 10 participating sites. MRI (blue) was the most common imaging across all sites, while the PSMA PET scan (yellow) was the least common. CT (green) and bone scans (orange) varied depending on sites.

The study cohort comprised 13,122 patients, categorized into the following four risk groups: 3502 (27%) classified as low risk, 2364 (18%) as favorable intermediate risk, 3585 (27%) as unfavorable intermediate risk, and 3671 (28%) as high risk. Figure 1 summarizes the distribution of imaging studies obtained. Among the low-risk cohort, the predominant imaging modality utilized was pelvic MRI, which was performed in 2091 patients (59.7%). Additionally, conventional bone scans were conducted in 622 patients (17.8%), CT scans in 562 patients (16.0%), and PET-based imaging in 17 patients (0.5%). 

A total of 718 patients underwent an MRI along with an additional imaging test. Specifically, 415 patients received both an MRI and a bone scan, 290 patients had an MRI and a CT scan, and 13 patients underwent an MRI and a PSMA PET scan. Among the patients who received a CT scan, 290 also had a bone scan, and 6 were additionally imaged with a PSMA PET scan. Furthermore, eight patients who had bone scans also underwent PSMA PET imaging. 

Figure 1 highlights the variability across the 10 participating sites. MRI emerged as the most frequently used imaging modality for low-risk prostate cancer patients. However, there was considerable variability in its usage among the sites from 29.5% to 94.2%. The use of CT scans varied between 5.7% and 22.3%, while bone scan utilization ranged from 0% at one site to as high as 33.3% at others. Despite its limited utility in low-risk prostate cancer cases, PSMA PET scan usage also showed variability, ranging from 0% to 2.2%.

**Figure 2:**
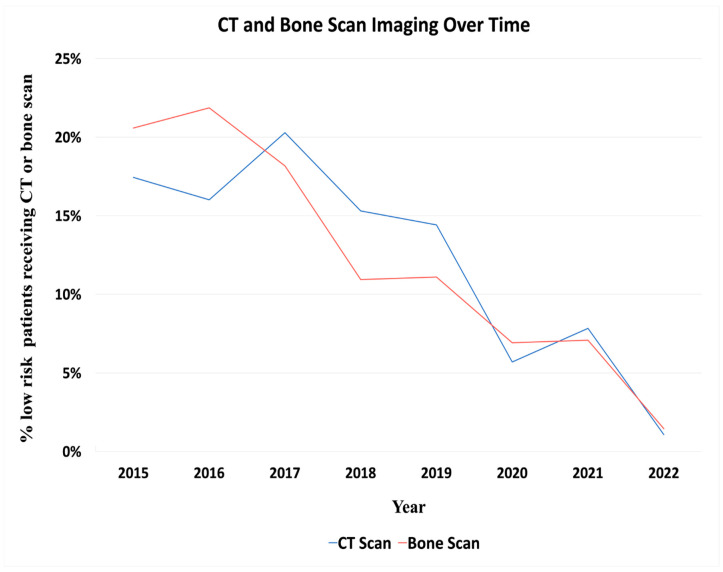
Distribution of total CT (Blue) and bone scans (Red) throughout the study period. The majority of CT and bone scans were obtained early in the study, with a reduction to 1.0% for CT scans and 1.5% for bone scans by 2022.

Figure 2 depicts the trend in the use of CT scans and bone scans throughout the study period. The percentage of patients receiving CT scans decreased from 17.4% in 2015 to 1.0% in 2022. Similarly, the use of bone scans declined from 20.6% to 1.5% over the same period.

## 4. Discussion

Our study found that MRI is the most commonly used imaging modality for men with low-risk prostate cancer. It is important to note that these MRI are primarily utilized for biopsy guidance and lesion identification rather than for whole-body imaging and staging. This aligns with the need for precise surgical planning in this patient group. Interestingly, we observed that a significant number of patients with low-risk prostate cancer were being staged with CT scans (24.5% of the cohort), bone scans (18.0% of cohort), and, in rare cases, PSMA PET scans (0.5% of the cohort). These practices are contrary to the current AUA guidelines and recommendations, which advise against such extensive imaging in low-risk cases. 

Redundant imaging remains prevalent, with 1022 patients (29.2%) undergoing multiple imaging modalities. This indicates a substantial deviation from the guideline-based care and highlights the need for continued efforts to optimize imaging practices in this population. As highlighted in Figure 2, the portion of patients receiving CT and bone scans have decreased over time, a trend likely attributable to iterative educational efforts. Physicians from the practices within PURC regularly meet to analyze data and trends, collaboratively developing best practices and practice patterns. These continuous educational interventions have likely contributed to the observed reduction in the use of CT and bone scans over the study period. 

Our study aligns with the goals of the Choosing Wisely campaign, which aims to reduce inappropriate imaging for men with low-risk prostate cancer. Since the initiation of this campaign, there has been a notable decline in the use of bone scans and CT imaging for staging newly diagnosed low-risk prostate cancer [10]. Discouraging unnecessary imaging tests not only reduces wasteful testing but also alleviates the financial burden associated with downstream care that may not offer substantial benefits to patients [9]. This trend is reflected in our findings, where continuous educational interventions and regular analysis of data by the PURC physicians contributed to a reduction in CT and bone scan utilization over time. Despite this progress, redundant imaging remains prevalent, with 1022 patients (29.2%) undergoing multiple imaging modalities. This indicates a substantial deviation from guideline-based care and underscores the need for ongoing efforts to optimize imaging practices in this population.

Low-risk prostate cancer management has evolved to prioritize the use of MRI over CT scans or bone scans due to the superior diagnostic capabilities of MRI in this context. Studies have consistently shown that CT scans are not necessary for low-risk prostate cancer patients, as they offer limited benefits in detecting disease progression or metastases in this population [11]. MRIs provide detailed imaging of the prostate gland, enabling the accurate visualization of tumors and aiding in treatment planning decisions [12]. It is particularly effective in guiding targeted biopsies, assessing tumor aggressiveness, and determining the need for active surveillance, surgery, or radiotherapy in low-risk prostate cancer patients [12]. The MRI utilization rate of 60% in patients who were stratified to the low-risk group could be explained by patients with contraindications to MRI and urologists still in the process of shifting practice from trans-rectal ultrasound biopsy to MRI-targeted biopsy. This shift toward MRIs aligns with our study’s findings and supports the Choosing Wisely campaign’s goals to reduce inappropriate imaging. Our data indicated that MRIs were the most prevalent imaging across all sites.

A major strength of our study is its alignment with other findings that emphasize the prioritization of MRIs for low-risk prostate cancer management. Our data are consistent with previous studies, reinforcing that MRI remains the most commonly used imaging modality, while the use of CT and bone scans has decreased. Comprehensive data from a large regional quality collaborative enhances the generalizability of our results, providing a real-world snapshot of imaging practices and trends over a significant period.

However, our study also has several limitations. Being a retrospective analysis, it is subject to the inherent limitations of such studies. The data were extracted from a database, which may contain inaccuracies or incomplete entries that could affect the reliability of our findings. The variability in data recording practices across different sites may also introduce inconsistencies. Additionally, in certain instances, a PSMA PET scan may have been used as an alternative to a prostate MRI, for example, when a patient has an implanted device that is not MRI compatible, and this may account for some instances of PET utilization in our study [13]. Despite these limitations, the large sample size and the extended study period provide valuable insights into imaging practices for low-risk prostate cancer, highlighting areas for improvement and the impact of ongoing educational initiatives.

## 5. Conclusions

Low-risk prostate cancer accounted for approximately 25% of new diagnoses within this large collaborative. Despite guidelines advising against extensive imaging for low-risk patients, approximately 15% of these patients underwent staging imaging using either CT or bone scans. Additionally, redundant imaging occurred in almost one-third of the men, indicating a substantial deviation from recommended practices. These findings underscore the critical need for continued education based on established guidelines to optimize resource stewardship and reduce unnecessary costs to the healthcare system.

Expanding efforts to educate both clinicians and patients about the appropriate use of imaging modalities could further align practices with current recommendations, ultimately enhancing the quality of care. Future research should focus on identifying the barriers to adherence to the imaging guidelines and developing strategies to address these challenges. By continuing to refine and disseminate best practices, we can improve patient outcomes and achieve more cost-effective care for low-risk prostate cancer patients.

## Data Availability

Data were provided with permission from the Pennsylvania Urologic Regional Collaborative (PURC) participating urology practices. PURC is a quality improvement initiative led by the Health Care Improvement Foundation which brings urology practices together in a physician-led data sharing and improvement collaborative aimed at advancing the quality of diagnosis and care for men with prostate cancer.

## References

[1] Merdan S., Womble P.R., Miller D.C., Barnett C., Ye Z., Linsell S.M., Montie J.E., Denton B.T. (2014). Toward better use of bone scans among men with early-stage prostate cancer. Urology.

[2] Risko R., Merdan S., Womble P.R., Barnett C., Ye Z., Linsell S.M., Montie J.E., Miller D.C., Denton B.T. (2014). Clinical predictors and recommendations for staging computed tomography scan among men with prostate cancer. Urology.

[3] Makarov D.V., Trock B.J., Humphreys E.B., Mangold L.A., Walsh P.C., Epstein J.I., Partin A.W. (2007). Updated nomogram to predict pathologic stage of prostate cancer given prostate-specific antigen level, clinical stage, and biopsy Gleason score (Partin tables) based on cases from 2000 to 2005. Urology.

[4] Courtney P.T., Deka R., Kotha N.V., Cherry D.R., Salans M.A., Nelson T.J., Kumar A., Luterstein E., Yip A.T., Nalawade V. (2022). Metastasis and Mortality in Men With Low- and Intermediate-Risk Prostate Cancer on Active Surveillance. J. Natl. Compr. Canc. Netw..

[5] Eastham J.A., Auffenberg G.B., Barocas D.A., Chou R., Crispino T., Davis J.W., Eggener S., Horwitz E.M., Kane C.J., Kirkby E. (2022). Clinically Localized Prostate Cancer: AUA/ASTRO Guideline, Part I: Introduction, Risk Assessment, Staging, and Risk-Based Management. J. Urol..

[6] Alshehri S.Z., Safar O., Almsaoud N.A., Al-Ghamdi M.A., Alqahtani A.M., Almurayyi M.M., Autwdi A.S., Al-Ghamdi S.A., Zogan M.M., Alamri A.M. (2020). The role of multiparametric magnetic resonance imaging and magnetic resonance-guided biopsy in active surveillance for low-risk prostate cancer: A systematic review. Ann. Med. Surg..

[7] Lange S.M., Choudry M.M., Hunt T.C., Ambrose J.P., Haaland B.A., Lowrance W.T., Hanson H.A., O’Neil B.B. (2023). Impact of choosing wisely on imaging in men with newly diagnosed prostate cancer. Urol. Oncol..

[8] Schnipper L., Smith T., Raghavan D., Blayney D., Ganz P., Mulvey T., Wollins D. (2012). American society of clinical oncology identifies five key opportunities to improve care and reduce costs: The top five list for oncology. J. Clin. Oncol..

[9] Makarov D.V., Loeb S., Ulmert D., Drevin L., Lambe M., Stattin P. (2013). Prostate cancer imaging trends after a nationwide effort to discourage inappropriate prostate cancer imaging. JNCI J. Natl. Cancer Inst..

[10] Pettit S., Mikhail D., Feuerstein M. (2022). Systematic review of interventions that improve provider compliance to imaging guidelines for prostate cancer. Can. Urol. Assoc. J..

[11] Prasad S.M., Gu X., Lipsitz S.R., Nguyen P.L., Hu J.C. (2011). Inappropriate utilization of radiographic imaging in men with newly diagnosed prostate cancer in the united states. Cancer.

[12] Shao W., Bhattacharya I., Soerensen SJ C., Kunder C.A., Wang J.B., Fan R.E., Ghanouni P., Brooks J.D., Sonn G.A. Weakly supervised registration of prostate mri and histopathology images. Proceedings of the Medical Image Computing and Computer Assisted Intervention–MICCAI 2021: 24th International Conference.

[13] Pepe P., Pepe L., Cosentino S., Ippolito M., Pennisi M., Fraggetta F. (2022). Detection Rate of 68Ga-PSMA PET/CT vs. mpMRI Targeted Biopsy for Clinically Significant Prostate Cancer. Anticancer. Res..

